# Case Report: Mineralized Pulmonary Artery Thrombi in Two Dogs Treated for Meningoencephalitis of Unknown Origin

**DOI:** 10.3389/fvets.2020.569597

**Published:** 2020-12-17

**Authors:** Suzanne Rosen, Leontine Benedicenti, Scott Petesch, Jennifer Reetz, Evelyn Marie Galban

**Affiliations:** School of Veterinary Medicine, University of Pennsylvania, Philadelphia, PA, United States

**Keywords:** meningoencephalitis of unknown etiology (MUE), meningoencephalitis of unknown origin, pulmonary arterial thromboembolism, cytosine arabinoside (ara-C), prednisone side effects

## Abstract

Meningoencephalitis of unknown origin (MUO) is a relatively common and very serious canine neurologic condition, which is typically associated with a poor long term prognosis despite treatment. This case series chronicles two dogs diagnosed with MUO who were treated with long term corticosteroids and cytosine arabinoside and lived well-beyond the typical survival time for this condition. Both eventually succumbed to respiratory signs associated with mineralized thrombi in their pulmonary arteries. Adverse effects from the two drugs used for treatment are reviewed in order to propose a possible mechanism to explain how long term use of these medications could result in such a phenomenon.

## Introduction

Meningoencephalitis of unknown origin (MUO) is an idiopathic inflammatory condition of the central nervous system that results in progressive neurologic dysfunction in dogs. Prognosis for animals with MUO tends to be guarded to poor with median survival times on the order of days to weeks (1–3). Many treatment protocols exist with one of the most successful being a long, tapering course of corticosteroids (initial prednisone dose of 1–2 mg/kg PO BID) in combination with cytosine arabinoside (CA) injections (50 mg/m^2^ SQ q12h for 4 doses every 3–6 weeks). Even with this protocol, documented median survival time only ranges from 26–531 days (1, 3, 4). Therefore, longitudinal studies associated with MUO, including the side effects of prolonged use of this medication combination, are lacking.

The present case series details the clinical course of two dogs diagnosed with MUO who were treated with long term corticosteroids and cytosine arabinoside injections. Both developed pronounced respiratory distress as well as similar radiographic abnormalities suggestive of mineralization in the pulmonary arteries. We propose that extended use of corticosteroids and cytarabine may be associated with these mineralized thrombi and subsequent respiratory signs, a complication which, to our knowledge, has not been previously reported.

## Case Presentation

### Case 1

An 8-month-old female-spayed Cavalier King Charles Spaniel dog presented for evaluation of recent seizure activity and mentation change. A few days prior, the dog had been evaluated by her primary care veterinarian for altered mentation. The referring veterinarian reported abnormal behavior and an elevated rectal temperature and initiated treatment with fluids, non-steroidal anti-inflammatories, and antibiotics. After the primary care visit, the owner observed two additional seizure-like events, which prompted referral.

**Figure 1:**
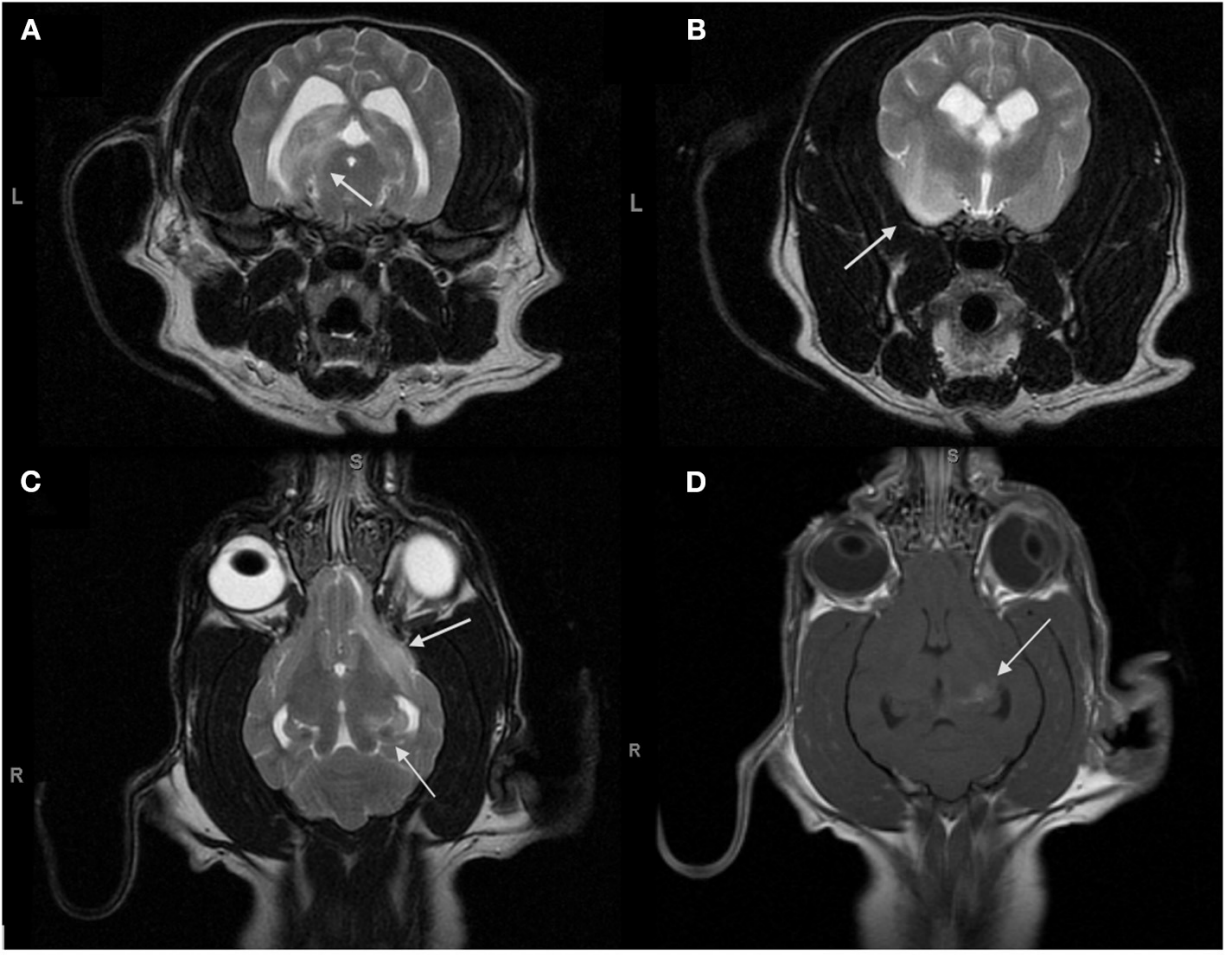
Magnetic resonance transverse T2-weighted images (T2Wi) **(A,B)**, dorsal T2Wi **(C)** and dorsal T1W with gadolinium **(D)** of the brain obtained for the initial diagnosis for MUO. **(A–C)** Multifocal to coalescing regions of hyperintensity on T2W are present in the left cerebral hemisphere and involve the left ventral frontal lobe, the left pyriform lobe, the left temporal lobe, and the left hippocampus. **(D)** The central region of the hippocampus enhances strongly with gadolinium while the remainder of the lesions do not enhance.

Upon presentation, general physical examination was unremarkable. Neurologic examination revealed a dull mentation, bilateral cortical blindness, decreased direct pupillary light reflex (PLR) in the left eye with a decreased consensual PLR in the right eye as well as mild general proprioceptive ataxia affecting all four limbs–indicating a multifocal intracranial neurolocalization. During hospitalization, concern arose for elevated intracranial pressure and Cushing reflex, as the heart rate dropped from 100 to 72 bpm, while the blood pressure rose from 130 to 185 mmHg. Treatment with a mannitol infusion (0.5 g/kg over 30 min) was initiated. On neurological examination the next day, strabismus, horizontal nystagmus with a fast phase to the left, and a focal seizure were observed, consistent with the multifocal neurolocalization. The hematology and serum biochemistry were within the reference ranges. The dog underwent general anesthesia (induced with diazepam 0.25 mg/kg IV and thiopental 10 mg/kg IV and maintained on a propofol CRI at 0.1 mg/kg/min) and an MRI of the brain^1^ and C1-C2 spinal segments was performed. The following imaging sequences were obtained: sagittal T2-weighted (T2W); transverse T2W, T2^*^-gradient echo, T2W fluid attenuated inversion recovery, T1-weighted (T1W), T1W following IV gadolinium administration, diffusion-weighted, and apparent diffusion coefficient map; and dorsal T1W and T1W with contrast (Figure 1).

The MRI revealed multifocal to coalescing lesions of T2W hyperintensity in the left cerebral hemisphere, involving the left frontal, temporal, piriform lobes, and the left hippocampus. The lesions were all slightly hypointense on T1W images, with the exception of the central area of the hippocampal lesion. This area of T1W hyperintensity was also the only region to contrast enhance. Moderate intramedullary hyperintensity at the level of C1–C2 on T2W was also reported. A sample of cerebrospinal fluid was collected from the cisterna magna. Analysis of the cerebrospinal fluid revealed a mononuclear pleocytosis with an elevated total protein of 119 mg/dL (reference interval (RI) <40 mg/dL), an elevated nucleated cell count of 49 cells/ uL (RI <10–18 cells/ uL) and an elevated RBC count of 423 cells/uL (RI <0–5 cells/ uL). Serum antibody titers for Rocky Mountain spotted fever, Neospora caninum, and Toxoplasma gondi were negative. Based on the multifocal hyperintense T2W MRI lesions as well as the hypercellular, predominantly mononuclear CSF, meningoencephalitis of unknown origin (MUO) was diagnosed, with granulomatous meningoencephalitis (GME) being the most likely form (5).

The dog was hospitalized in the ICU and treated with immunosuppressive therapy consisting of corticosteroid injections (dexamethasone SP 0.1 mg/kg IV q24) and a cytarabine constant rate infusion (200 mg/m^2^ over 24 h). Loading doses of phenobarbital (4 mg/kg IV q6 for 24 h) were also administered. Over the next few days, despite persistent visual deficits, the patient showed resolution of nystagmus and seizure activity and improvement in mentation. The dog was discharged on prednisone (1 mg/kg PO BID) and phenobarbital (2 mg/kg PO BID), with instructions to return every 3–5 weeks for subsequent cytarabine injections.

Over the next four and a half years, eighteen total courses of cytarabine (200 mg/m^2^ SQ q12 over 4 doses) were administered. During the first year of treatment, prednisone was gradually tapered and then discontinued from the starting dose due to intolerable side effects. In the following years, prednisone was restarted twice at the initial dose due recurrence of her original neurologic abnormalities and seizure activity. Each time it was slowly tapered over a few months due to side effects. Adjunctive treatments were utilized based on response to therapy and medication side effects. Phenobarbital was tapered from the starting dose of 2 mg/kg PO BID over a 9-month period. It was restarted shortly thereafter at the initial dose due to worsening neurologic signs and then weaned over a year and a half. A few months into treatment, in response to two seizure episodes, cyclosporine was started at a dose of 7 mg/kg PO daily and then slowly weaned over a 2-year period. At this same visit, potassium bromide was also added at a starting dose of 20 mg/kg PO daily and continued throughout life with the daily dosage varying from approximately 4.5 mg/kg PO to 37.5 mg/kg PO. About 2 years later, after several seizure episodes, levetiracetam was introduced at a dose of 22.5 mg/kg PO TID and maintained at dosages varying from 7.5 mg/kg TID to 30 mg/kg PO TID. A few months later, following an additional seizure event, zonisamide was started and maintained at a dose of 6 mg/kg PO BID.

**Figure 2:**
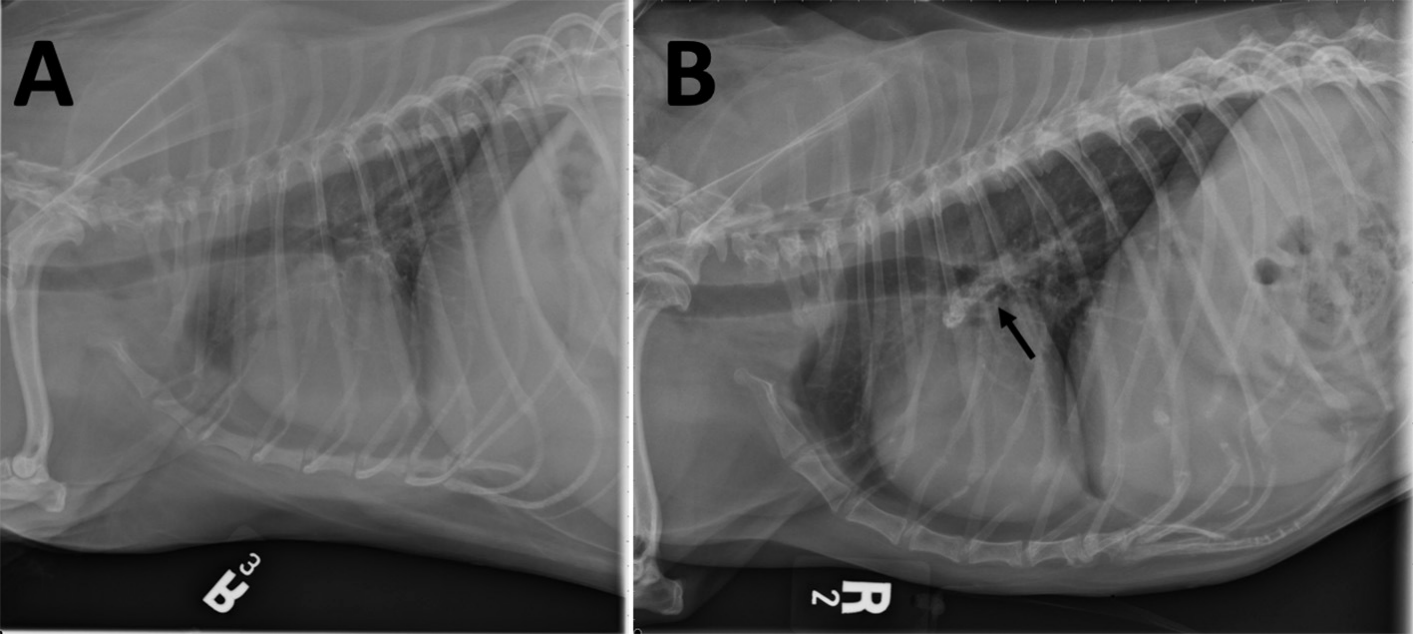
Right lateral thoracic radiographs **(A)** prior to treatment and **(B)** after 4 years of treatment for MUO. **(B)** Mineralization in the right and left pulmonary arteries (arrow) is seen as an elongated heterogeneous mineral opacity overlying and extending slightly dorsal and ventral to the carina. This finding is not evident in **(A)**.

**Figure 3:**
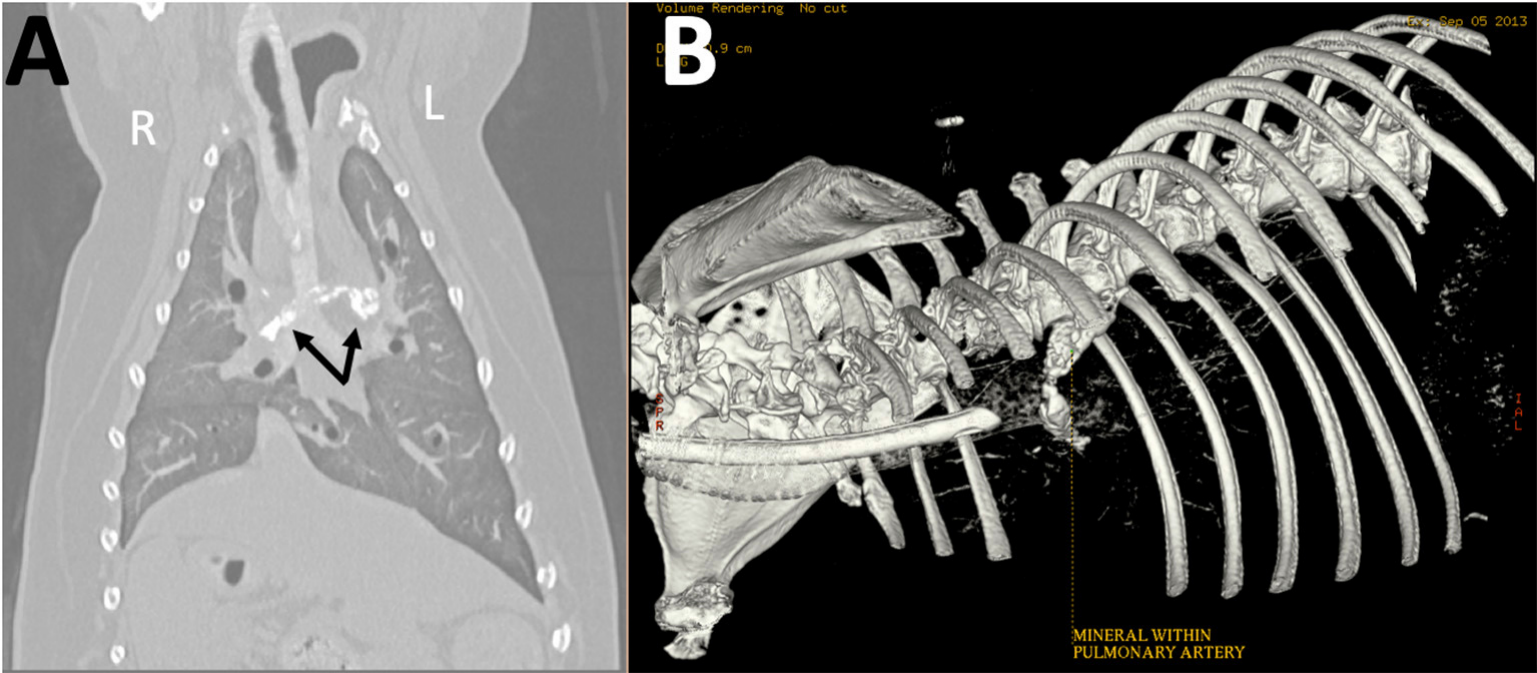
Reconstructed **(A)** dorsal and **(B)** three-dimensional (3D) computed tomographic images obtained post-mortem show amorphous mineral attenuation within the right and left pulmonary arteries (arrows).

Five years after the original diagnosis, the dog presented to the emergency service with the presenting complaint of a few day history of coughing, lethargy, and inappetence. Chest radiographs revealed a large mineral opaque tubular structure in the region of the pulmonary artery (Figure 2). The owner elected euthanasia after the patient failed to respond to supportive therapy. Post-mortem computed tomography (CT) of the chest as well as an necropsy were performed. The CT confirmed that there was a mineralized thrombus occupying the entire lumen of both the right and left pulmonary arteries, causing near complete blockage (Figure 3). Pathology revealed a chronic thrombus (6 cm long × 0.8 cm in diameter) with extensive mineralization adhered to the pulmonary trunk and extending 2.5 cm into the right and left pulmonary arteries. Histopathology of the brain revealed mild left cerebral and thalamic atrophy with mild hydrocephalus ex vacuo as well as extensive left hippocampal parenchymal loss with astrocytic sclerosis.

### Case 2

A 7-year-old female-spayed Pekingese dog presented for evaluation of acute onset of weakness, anorexia, inability to walk, and a seizure-like event. The dog had previously been diagnosed with thoracolumbar and mild caudal cervical intervertebral disc disease (IVDD) based on MRI and myelogram results which, for the past year, was being medically managed with prednisone and rest. General physical examination was unremarkable. Neurologic examination revealed a non-ambulatory tetraparesis with severe proprioceptive deficits. The patient also had a left-sided head tilt and horizontal nystagmus with a fast phase to the right as well as bilateral cortical blindness. These deficits were consistent with a multifocal intracranial neurolocalization and the dog was admitted to the hospital for further diagnostics and monitoring.

Hematology and serum biochemistry revealed a mildly elevated ALT of 333U/L (16–91 U/L) as well as a mild neutrophilia of 16.39 × 10^3^/ul (3.1–14.4 × 10^3^/ul) and lymphopenia of 0.51 × 10^3^/ul (0.9–5.5 × 10^3^/ul). Fasting serum ammonia level and fasting bile acids were within the normal reference ranges. The dog underwent general anesthesia (drugs and doses unknown). MRI of the brain revealed multifocal brain disease, however the MR images and official radiology report are unavailable for review. CSF analysis revealed a mixed pleocytosis with an elevated total protein of 277 mg/dL (RI <40 mg/dL), an elevated nucleated cell count of 277 cells/ uL (RI <10–18 cells/ uL) and an elevated RBC count of 189 cells/uL (RI <0–5 cells/ uL). Antibody titers were negative for Neospora caninum, Toxoplasma gondi, Ehrlichia canis, and equi and Rocky Mountain spotted fever. MUO was diagnosed based on these results.

Dexamethasone SP injections (0.2 mg/kg IV q12) and a cytarabine constant rate infusion (200 mg/m^2^ IV over 24 h) were administered. Loading doses of phenobarbital (4 mg/kg IV q6 for 4 doses) were also initiated. A few days later, the patient showed improved proprioception and menace response bilaterally. The left-sided head tilt and horizontal nystagmus resolved. The dog was discharged on prednisone (1 mg/kg PO BID) and phenobarbital (2 mg/kg PO BID). Cytarabine injections were scheduled to continue every 3–5 weeks.

Over the next 6 years, a total of forty-six courses of cytarabine injections (200 mg/m^2^ SQ q12 over 4 doses) were administered. Due to side effects of the prednisone, the dose was gradually tapered from the initial starting. However, each time it was completely discontinued, the dog's neurologic deficits worsened, therefore a low dose of prednisone was maintained. Months into treatment, severe interscapular calcinosis cutis and deep pyoderma were observed. The pyoderma resolved with cephalexin (22 mg/kg PO q12) but the calcinosis cutis progressed to encompass the entire dorsum and part of the inguinal area. Decreasing the dose of prednisone helped resolve the skin issues, however, every attempt to discontinue corticosteroids lead to return of neurologic signs. Additional medications were utilized to help limit medication side effects as well as neurologic signs. Over the first 6 months of treatment, phenobarbital was tapered and then discontinued from the initial dose of 2 mg/kg PO BID. Around the time phenobarbital was stopped, mycophenolate was introduced and then maintained for 2 years at 10 mg/kg PO BID. About 1 year into treatment, azathioprine was added due to continued steroid side effects at 1 mg/kg PO daily. It was tapered and then discontinued over 4 years.

**Figure 4:**
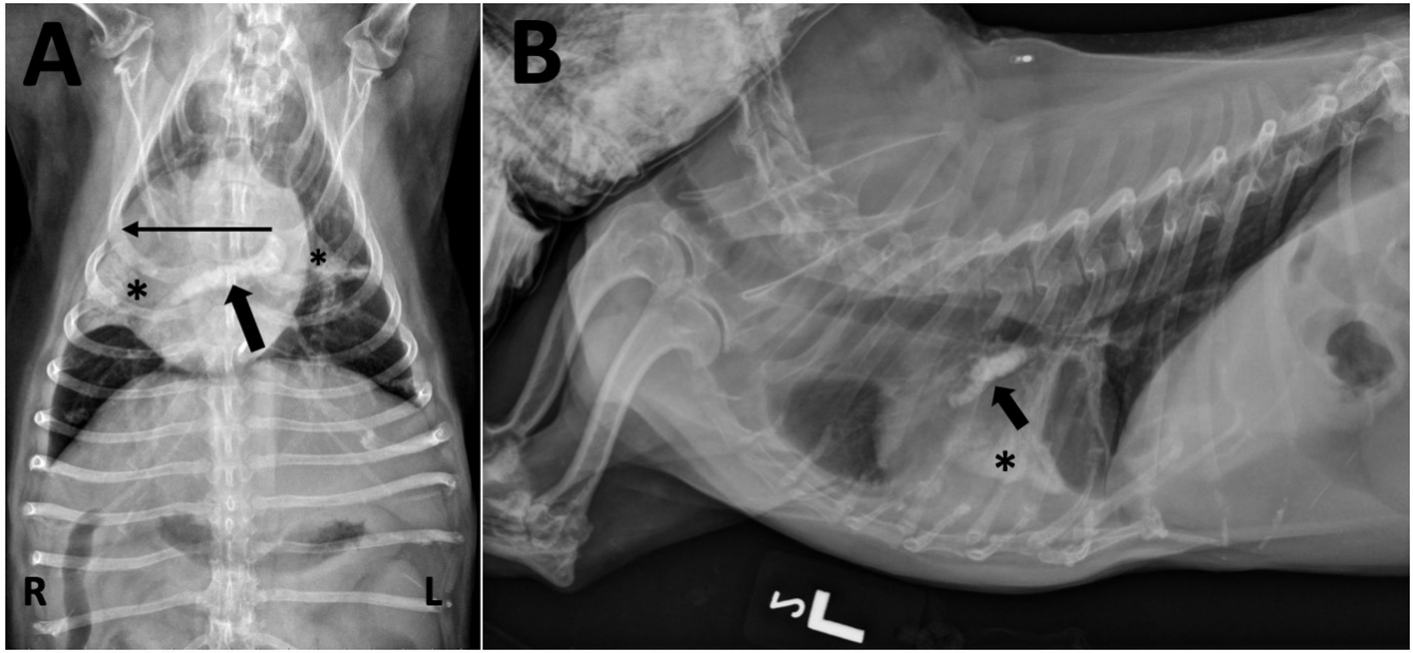
**(A)** Ventrodorsal and **(B)** left lateral thoracic radiographs taken after 5 years of treatment for MUO. **(A)** A tubular mineral opacity is seen in the region of the right main and right caudal lobar pulmonary artery (black arrow). Mineral opacity and loss of volume is present in the right middle and caudal segment of the left cranial lung lobes (*) with an associated rightward mediastinal shift (long horizontal line). There are also air bronchograms visible within the right middle lobe. **(B)** The mineral opacity in the artery is seen ventral to the carina (black arrow); it may not seem to be extending caudally and dorsally (as expected for the right caudal lobar pulmonary artery) due to significant patient rotation and altered heart position from the mediastinal shift. The mineralized alveolar pattern with loss of volume in the right middle lung lobe is evident over the middle of the cardiac silhouette (*).

Five years after the original diagnosis, the dog presented for a recheck exam. A grade IV/VI right apical systolic murmur was auscultated for the first time. Difficulty breathing, significant weight loss and acutely worsening ataxia and paresis were also observed. Chest radiographs revealed mineralization in at least one pulmonary blood vessel, likely the right caudal lobar vessel, as well as calcification of the airways and lung parenchyma (Figure 4). Throughout the next year, the dog continued to have a mildly increased respiratory rate and effort as well as progressive pelvic limb weakness due to suspected orthopedic disease. Ultimately, euthanasia was elected and performed at her primary care veterinarian. Necropsy was not performed.

## Discussion

Prognosis for patients diagnosed with MUO is typically guarded to poor, with few canines living 2 years past diagnosis, even with an aggressive multimodal treatment approach (1, 3, 4). Therefore, limited research exists on the long-term complications of the medications, such as corticosteroids and cytarabine, used to treat this condition. This case series investigates two dogs with MUO who lived more than 4 years past diagnosis. Both patients were treated with prolonged oral corticosteroids and frequent cytarabine injections and eventually developed severe respiratory signs as well as radiographic evidence of pulmonary artery thrombus mineralization.

Although the exact etiology of MUO remains unknown, the condition is believed to be autoimmune in origin and therefore immunosuppression with corticosteroids, such as prednisone, is the traditional mainstay of treatment. In addition to pulmonary thrombus mineralization, one of the cases in the present study exhibited pulmonary parenchymal mineralization, which has been widely documented as a side effect of corticosteroid use in dogs. Both endogenous and iatrogenic hyperadrenocorticism have been associated with severe pulmonary mineralization in up to 90% of cases (6–9). The typical radiographic presentation of these patients is diffuse pulmonary mineralization characterized by a generalized interstitial pulmonary pattern and occasional mineralization of the tracheal rings and main-stem bronchi (10). The exact mechanism underlying this type of mineralization is not well-understood but it is hypothesized that high plasma cortisol concentration may alter the structure of or even catabolize collagen. Calcium, in turn, is believed to have a greater binding affinity for the organic matrix of these altered protein (6). Another well-documented sequela of hyperadrenocorticism is thrombus formation, including pulmonary thromboembolism (PTE) (11–13). Prednisone administration in healthy dogs has been directly associated with a hypercoagulable state, which may help explain this phenomenon (14, 15). Although pulmonary mineralization and PTEs have been separately documented to occur as side effects of prednisone, to our knowledge the patients depicted in this case series are the first to have presented with mineralized pulmonary thrombi. It is therefore unlikely that prednisone alone led to this complication and that another variable, which we postulate to be prolonged cytarabine use, was contributing.

Cytosine arabinase (cytarabine) is a chemotherapeutic agent used to treat a variety of cancers in humans and dogs. It quickly converts into a metabolite that damages DNA while the cell cycle is maintained in the S phase. Rapidly dividing cells are the most affected. Recently, cytarabine has been used as an adjunctive immunosuppressant agent to treat dogs with MUO due to its ability to cross the blood brain barrier. Some of the most well-documented and serious side effects of cytarabine use in human medicine are pulmonary toxicities (1, 16–19). It is hypothesized that cytarabine may damage pulmonary capillary endothelial cells due to its cytotoxic properties and thus lead to increased alveolar capillary permeability (16, 17). Side effects of cytarabine use in dogs have mainly been limited to myelosuppression and gastrointestinal distress (1). However, similar to reports in humans, there is a case of drug-induced infiltrative lung disease reported in a dog shortly after a cytarabine infusion. Like the dogs in our study, this dog had been diagnosed with MUO, was on long term prednisone and had received previous courses of cytarabine (20). In addition, a rare side effect of subcutaneous injections of cytarabine in dogs being treated with immunosuppressive doses of prednisone for MUO is the development of severe calcinosis cutis and deep pyoderma. The calcinosis cutis reported in these patients initially involved a focal interscapular region and progressed to encompass the whole dorsum and even the inguinal area. A very similar manifestation of calcinosis cutis and deep pyoderma was reported in one of the dogs in our case series. Interestingly, the original study reported that none of the MUO patients on prednisone monotherapy or dogs in the hospital on prednisone who received a subcutaneous injection of a different medication developed such skin reactions (21). Although the pathophysiology behind the development of this calcification remains undetermined, it may involve a similar mechanism to the thrombotic mineralization presented in this study.

Another less common complication of cytarabine's cytotoxic nature is severe vasculitis (22). This association is corroborated by *in vitro* research performed by Boogaret and associates, which revealed cytosine arabinoside's toxic effect on endothelial cells from human umbilical veins (23). The endothelium is vital in controlling hemostasis and damage to its cells has been associated with a pro-thrombotic state (24). Consequently, although not previously documented, it is possible that extended cytosine arabinoside use could result in pulmonary vascular endothelial damage and thereby act synergistically with the thrombotic and calcifying effects of prednisone to contribute to the formation of the mineralized thrombi observed in this case series.

This study demonstrates the importance of performing thoracic radiographs in order to serially monitor dogs on long term corticosteroids and cytarabine for pulmonary thrombotic mineralization. This serious complication has the potential to result in respiratory distress and death. Limitations in the present study include the small number of cases, absence of thoracic radiographs before the initiation of treatment as well as a lack of definitive pathology in one of the cases. A larger case study with preliminary as well as intermittent follow up chest radiographs and pathologic diagnoses is warranted.

## Data Availability Statement

The original contributions presented in the study are included in the article/Supplementary Material, further inquiries can be directed to the corresponding author/s.

## Ethics Statement

Ethical review and approval was not required for the animal study because this is a retrospective study using data previously collected on client-owned animals treated at our institution with their owners' consent. This study did not require recruitment of any animal for the research purpose and no procedure was performed for the sole purpose of this project. Written informed consent was obtained from the owners for the participation of their animals in this study.

## Author Contributions

SR examined cases and drafted the manuscript. LB, SP, and EG contributed to article conception as well as manuscript revision. The radiographs and CT image were reviewed and interpreted by JR. All authors contributed to the article and approved the submitted version.

## Conflict of Interest

The authors declare that the research was conducted in the absence of any commercial or financial relationships that could be construed as a potential conflict of interest.

## Footnotes

^1^Brivio MR355, 1.5 Tesla MRI unit, GE Healthcare, Milwaukee, Wis.
